# Supplementary material for: The MicroRNA Landscape of Acute Beta Cell Destruction in Type 1 Diabetic Recipients of Intraportal Islet Grafts
Source: Cells. 2021 Jul 4;10(7):1693. doi: 10.3390/cells10071693 (PMC8304265; doi:10.3390/cells10071693)
Supplement: Supplementary file 1 [file cells-10-01693-s001.zip › Supplementary 1.pdf]

## 1. Selection of reference microRNAs

For the selection of stable reference microRNAs, we calculated the standard deviation of all NRQ values (for all samples) for each microRNA. The microRNAs with the lowest NRQ standard deviation and that were detected in at least 15 of the total of 16 samples (*n* = 8, pre- and post-transplant) were imported in qbase+ Software v3.0 to determine the 15 best-ranked normalizers (geNorm M value). Selection of the optimal number of stable normalizers (geNorm V value) was based on geNorm s pairwise variation analysis between subsequent normalization factors using a cut-off value of 0.15 for the inclusion of additional normalizers. Finally, we checked the genomic location of the candidate reference microRNAs to avoid including microRNAs that are putatively co-regulated (> 2 kb).

In our discovery sample set, we selected 15 microRNAs with the lowest inter-sample variation (Figure S1 A), and these were further analyzed by geNorm, an algorithm based on reference gene ranking and stepwise elimination of the least stable gene (Figure S1 B). We calculated the internal control gene-stability measure M, defined as the average pairwise variation of a particular microRNA with all other control microRNAs where microRNAs with the lowest M values have the most stable expression [1]. Figure S1 A shows the M values for the 15 most stable microRNAs in our discovery cohort. These

15 microRNAs all show a superior stability (lower M value) to miR-16, a reference micoRNA widely used for data normalization. The three most stable microRNAs were miR-524-3p, miR-30e-3p and miR-208a-3p. To determine the minimum number of reference microRNAs that should be included for robust data normalization in our study, the pairwise variation $V_n/V_{n+1}$ was calculated between two sequential normalization factors ($NF_n$ and $NF_{n+1}$). A large variation means that the added gene has a significant effect and should preferably be included for calculation of a reliable normalization factor. We took a default cut-off value of 0.15, below which the inclusion of an additional control gene is not required. In our discovery data set, this analysis indicated that at least four microRNAs are required to accurately normalize

microRNAs present (Fig S1 B), with the four most stable microRNAs being miR-524-3p, miR-
30e-3p, miR-208a-3p and miR-1291. Absence of genomic co-localization of these microRNAs was verified to avoid possible co-regulation (chr19: 53711002-53711088, chr1: 40754355-40754446, chr14: 23388596-23388666 and chr12: 48654444-48654530 respectively) [2].

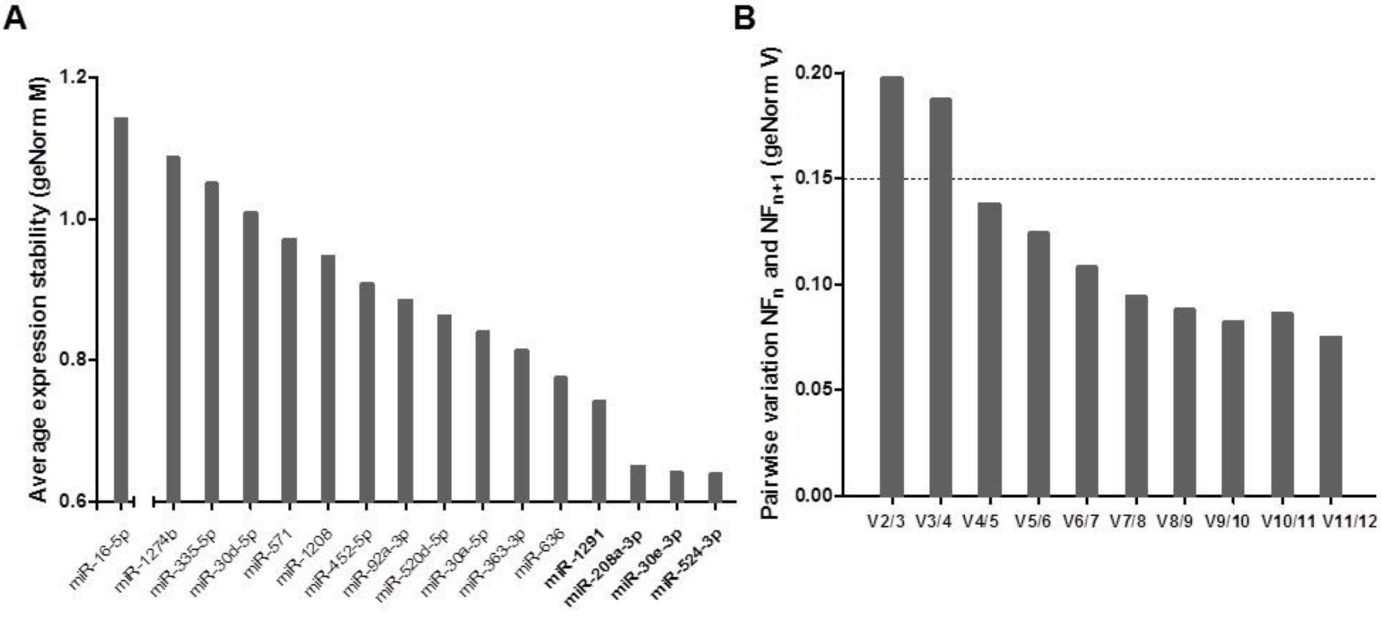


**Supplementary Figure St: Selection of a microRNA reference network for data normalization.** The 15 microRNAs with the lowest NRQ standard deviation (across all samples) were identified by the geNorm algorithm. **A.** Bar graphs indicate the average expression stability (geNorm M value) of the 15 most stable microRNAs in comparison to the M value of the commonly used reference microRNA miR-16-5p. **B.** Pairwise variation between two sequential normalization factors $NF_n$ and $NF_{n+1}$, where n stands for the number of references used, starting with the lowest M value. The cut-off value of 0.15 was used. Below this cut-off value, the inclusion of an additional control gene is not required. $V_n/V_{n+1}$ stands for the pairwise variation when n/n+1 reference microRNAs are used.

**2. Analysis of tissue selectivity of human microRNA expression**

The tissue tropism (relative expression level) of eight confirmed microRNA biomarkers of beta cell destruction was additionally verified in a public data repository, investigating microRNA expression 61 different human tissues or cell types [3].

## 2.1 Set of 4 confirmed microRNA biomarkers of beta cell destruction with an islet endocrine-enriched expression: miR-375, miR-132-3p, miR-204-5p and miR-410

**MiR-375** shows a very high cell-type selectivity (TSI > 0.9) with detectable expression in gastrointestinal organs and lungs but predominant expression in the brain, notably the pituitary gland. Low in the total pancreas.

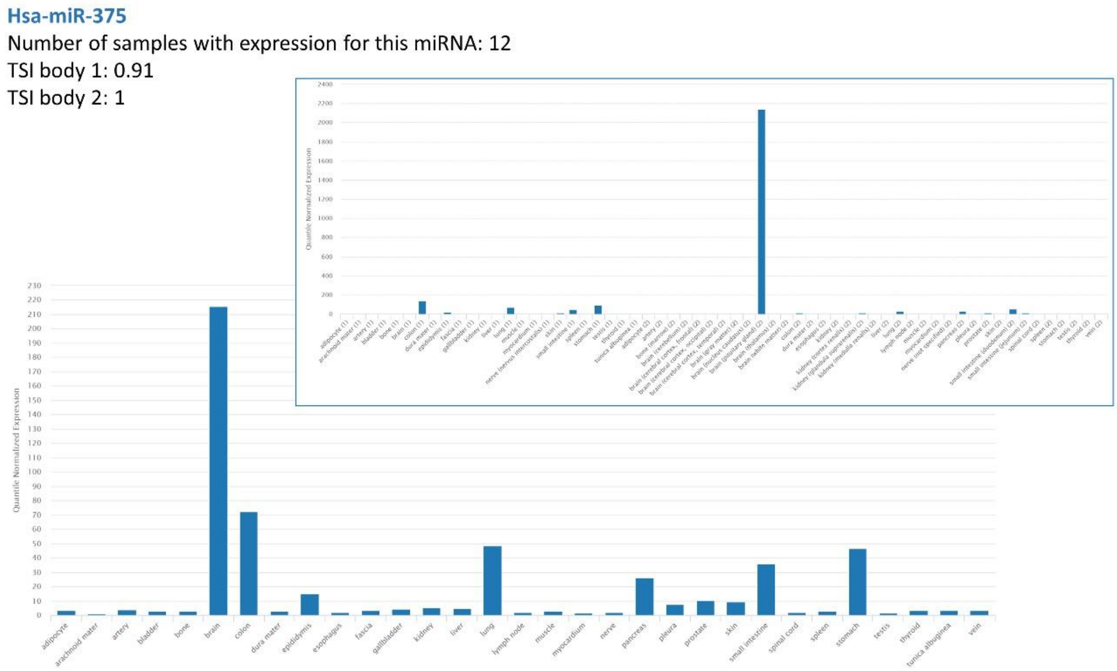


**MiR-132-3p** shows a very high cell-type selectivity (TSI > 0.9) with detectable expression in various organs but predominant expression in the brain, notably the pituitary gland. Low in the total pancreas.

**Hsa-miR-132-3p**

Number of samples with expression for this miRNA: 38

TSI body 1: 0.94

TSI body 2: 0.96

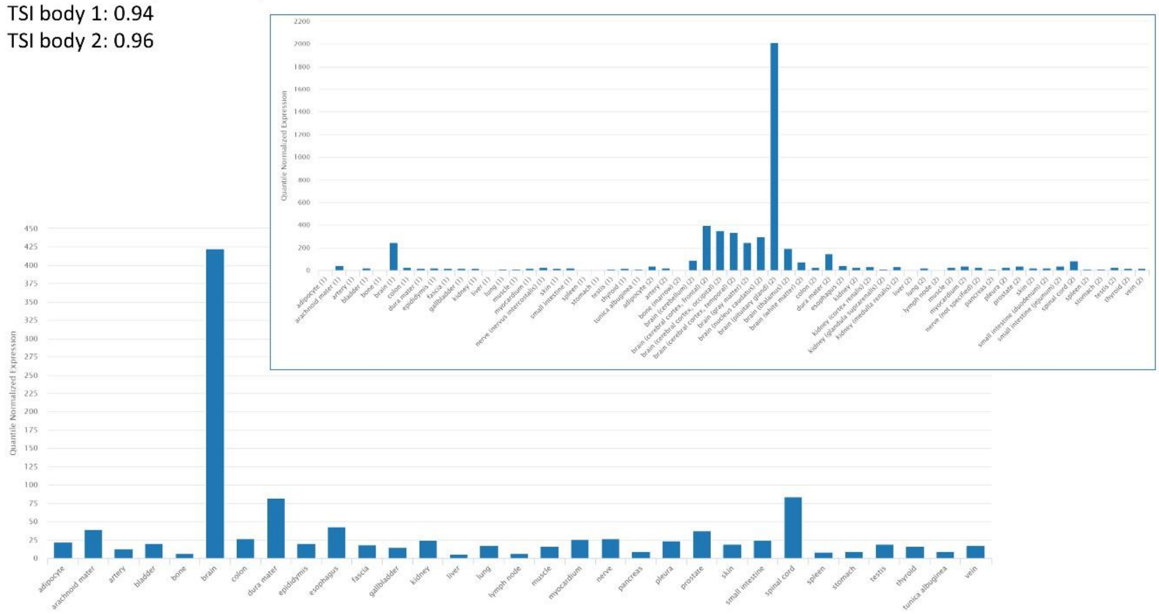


**MiR-204-5p** shows a high cell-type selectivity (TSI > 0.9) with detectable expression in various organs but predominant expression in the central nervous system, notably the cerebellum. Expression is relatively low in the pituitary gland. Low in the total pancreas.

**Hsa-miR-204-5p**

Number of samples with expression for this miRNA: 44

TSI body 1: 0.87

TSI body 2: 0.95

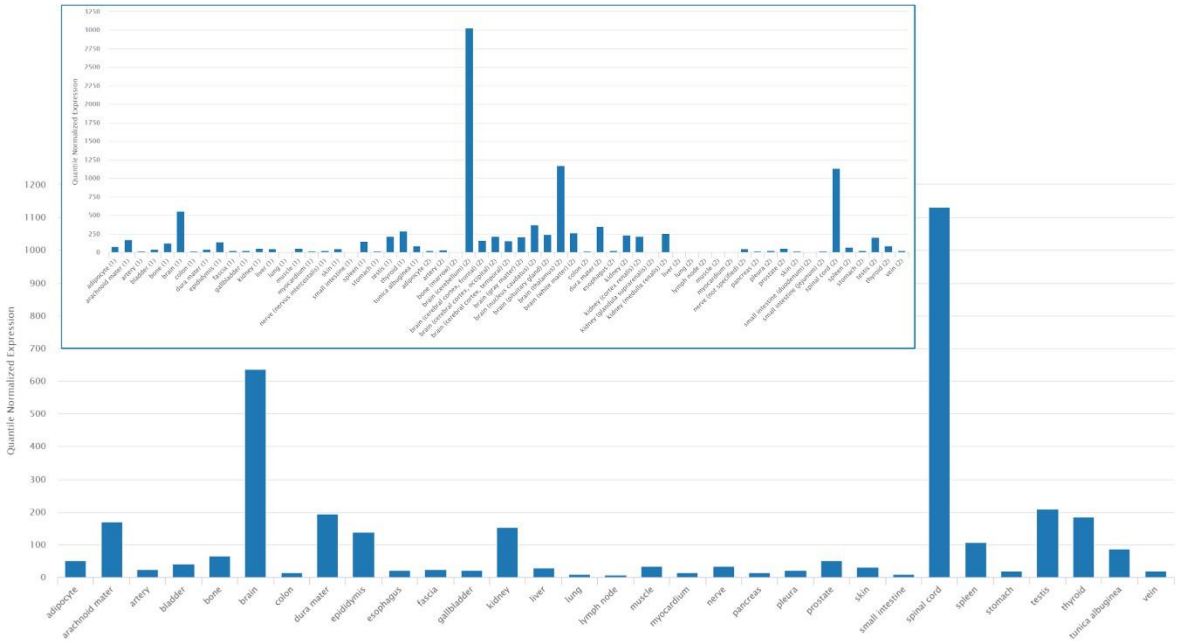

**MiR-410-3p** shows a very high cell-type selectivity (TSI > 0.9) with detectable expression in various organs but predominant expression in the brain, notably the pituitary gland. Low in the total pancreas.

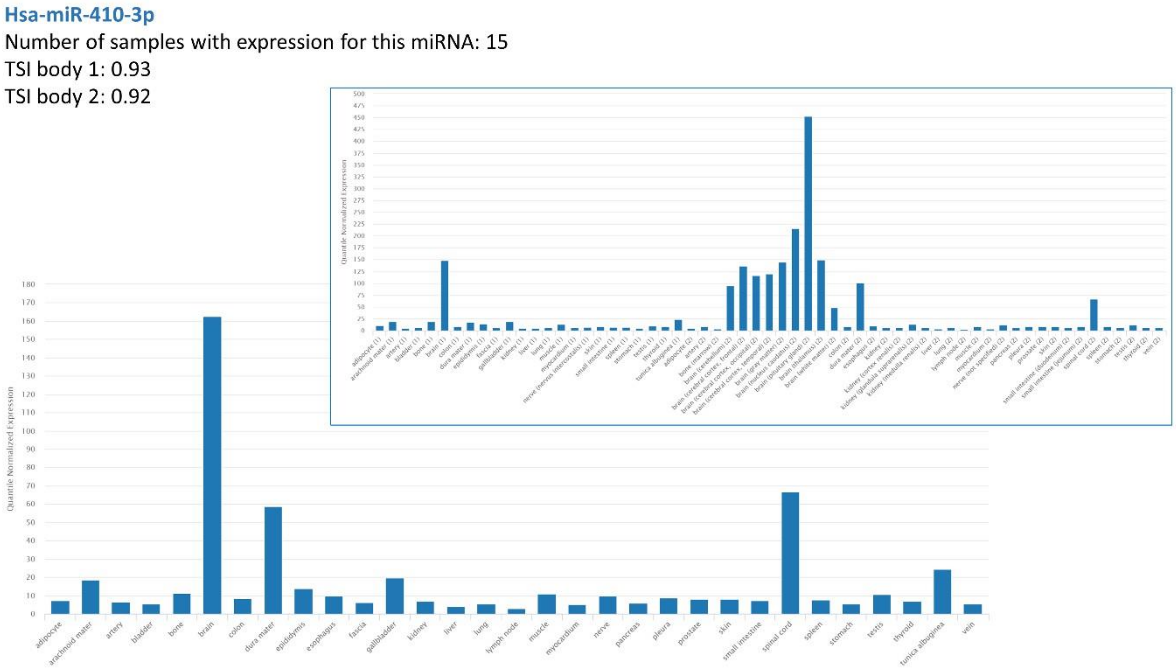


**2.2 Set of 4 confirmed microRNA biomarkers of beta cell destruction with an exocrine-enriched expression: miR-200a-3p, miR-429, miR-125b-5p, miR-216b-5p**

**MiR-410-3p and miR-429** show a high cell-type selectivity (TSI > 0.9) with detectable expression in various organs but predominant expression in the colon, skin, thyroid gland and pituitary gland. Low in the total pancreas.

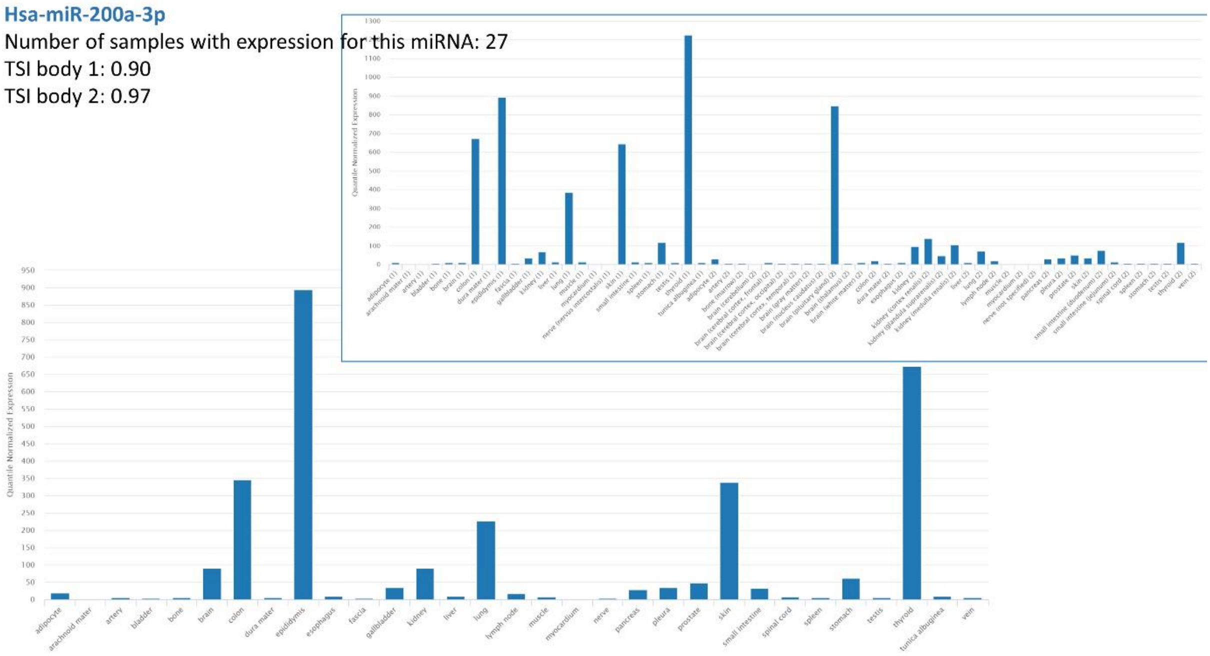

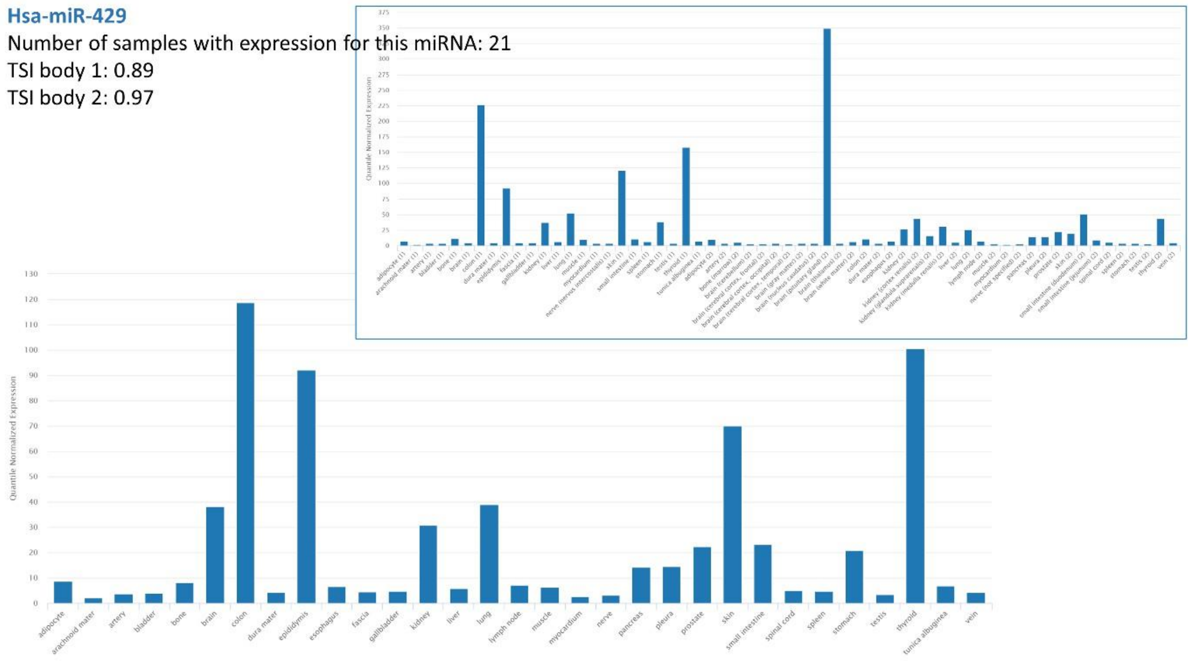


**MiR-125b-5p** shows a limited cell type selectivity (TSI <0.8) with detectable expression in many organs but predominant expression in brain regions.

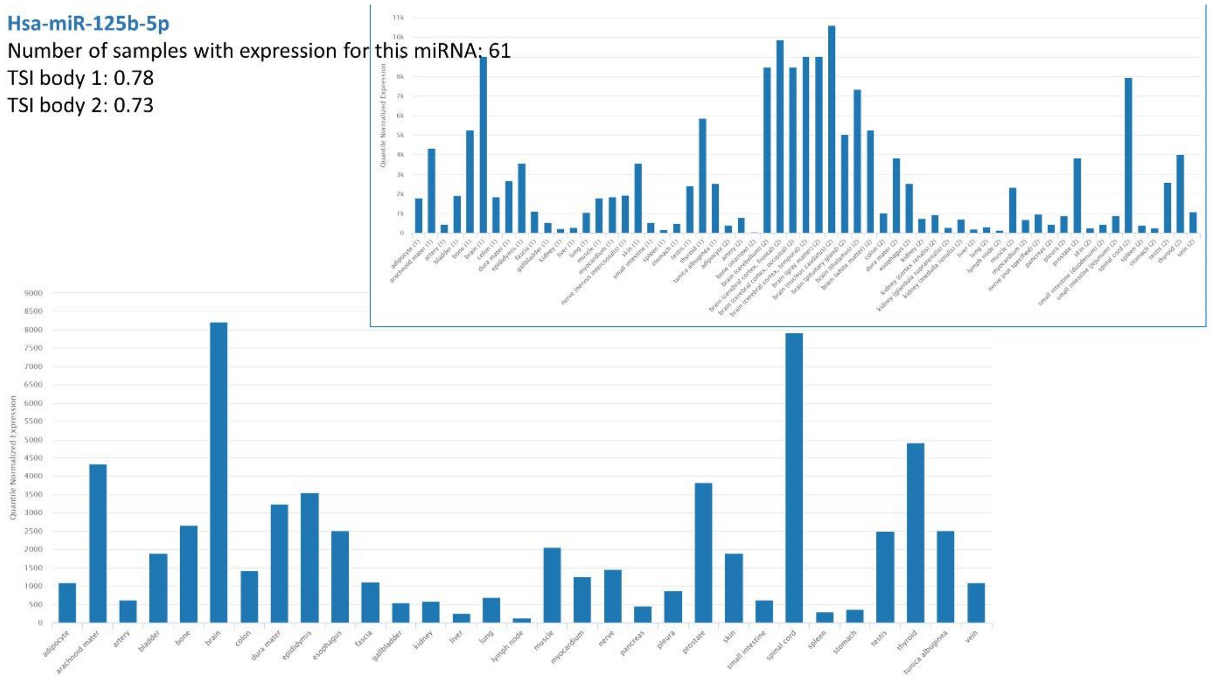


**MiR-216b-59** shows a high cell-type selectivity (TSI > 0.9) with detectable expression in various organs but predominant expression in the pancreas, compatible with exocrine cell selectivity.

## Hsa-miR-216b-5p

Number of samples with expression for this miRNA: 1

TSI body 1: 0.72

TSI body 2: 0.95

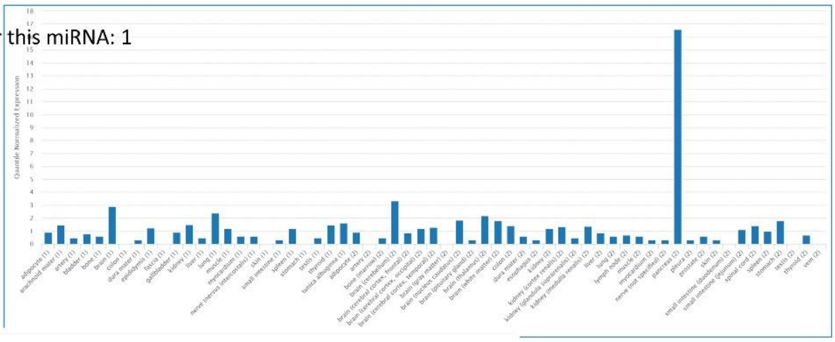

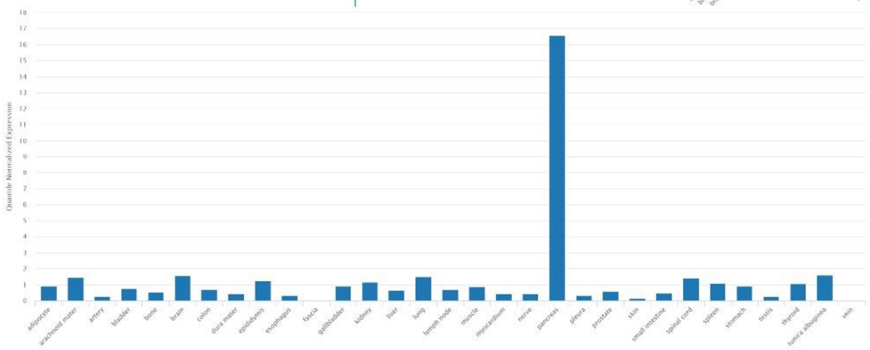

## References to supplementary material

1. Vandesompele J, De Preter K, Pattyn F, et al. Accurate normalization of real-time quantitative RT-PCR data by geometric averaging of multiple internal control genes. *Genome Biol* 2002; **3**(7): RESEARCH0034.

2. Megraw M, Sethupathy P, Corda B, Hatzigeorgiou AG. miRGen: a database for the study of animal microRNA genomic organization and function. *Nucleic acids research* 2007; **35**(Database issue): D149-55.

3. Ludwig N, Leidinger P, Becker K, et al. Distribution of miRNA expression across human tissues. *Nucleic acids research* 2016; **44**(8): 3865-77.
